# Mechanical rotational chair-assisted multiple canalith repositioning procedures for benign paroxysmal positional vertigo: enhanced vertigo relief, comparable adverse effects, and decreased incidence of residual dizziness

**DOI:** 10.3389/fneur.2023.1226138

**Published:** 2023-08-07

**Authors:** Hao Zhang, Meijia Zhu

**Affiliations:** ^1^Department of Neurology, Shandong Provincial Qianfoshan Hospital, Shandong University, Jinan, Shandong, China; ^2^Department of Neurology, The People's Hospital of Rizhao City, Rizhao, Shandong, China

**Keywords:** benign paroxysmal positional vertigo, multiple canalith repositioning procedures, mechanical rotational chair, adverse effects, residual dizziness

## Abstract

**Objectives:**

This retrospective study aimed to assess the effectiveness and adverse effects of mechanical rotational chair-assisted multiple canalith repositioning procedures (CRPs) to treat benign paroxysmal positional vertigo (BPPV).

**Materials and methods:**

A retrospective analysis of 1,273 BPPV patients was conducted, with 241 patients included in the final study. The participants diagnosed with BPPV, unresolved by a single previous CRP, were categorized into either the single or multiple CRP groups. In both groups, on days 1, 4, and 7 after the initial treatment, the participants were re-evaluated after a single CRP; if positional vertigo was resolved, the treatment was regarded as successful. The remission rate, adverse effects (such as canal switch (CS), falls, and vomiting), residual dizziness (RD) rate, and RD duration were compared between the two groups.

**Results:**

The resolution rates for the single and multiple CRP groups were significantly different on days 1 and 4 (55.7% vs. 85.1%, 75.5% vs. 91.9%; *P* < 0.05) but not on day 7 (93.3% vs. 94.8%; *P* > 0.05). There were no significant differences between the single and multiple CRP groups in terms of CS and falls (3.8% vs. 5.2%, 10.3% vs. 8.9%; *P* > 0.05). However, there was a significant difference in the incidence of vomiting (6.6% vs. 14.8%; *P* < 0.05). RD such as head heaviness, imbalance, and non-specific dizziness is more common in the single CRP group than in the multiple CRP group (34.9% vs. 20.7%, 42.5% vs. 26.7%, 47.2% vs. 32.6%; *P* < 0.05). The incidence and duration of RD were notably diminished in the group undergoing multiple CRPs compared to the single CRP group, with incidence rates of 41.5% and 57.5%, respectively (*P* < 0.05).

**Conclusion:**

For patients with BPPV, multiple CRPs offer greater benefits than a single CRP.

## Introduction

Benign paroxysmal positional vertigo (BPPV) is the most common type of vestibular vertigo, which is defined as paroxysms of vertigo caused by head position changes in the direction of gravity (1). As head position shifts, displaced otoconia traverse to the semicircular canals under gravitational influence, provoking internal lymphatic flow accompanied by a constellation of symptoms and signs, including vertigo, vomiting, and imbalance.

At present, CRP is the standard treatment for BPPV and has demonstrated significant therapeutic effects (2, 3). However, the superiority of multiple or single CRP within a single session for BPPV remains ambiguous until now. Some studies have reported that multiple CRPs in one session lead to an equal or higher resolution rate of positional vertigo and nystagmus than a single CRP (4–6). Although these studies have primarily evaluated the clinical benefits of multiple CRPs in treating BPPV, they have not yet explored the potential risks associated with multiple CRPs, including falls, CS, and RD. Some researchers have noted a certain degree of risk for re-entry into the semicircular canal as a consequence of retesting following CRM (7). RD has been detected in the majority of patients immediately following BPPV resolution, and the efficacy of multiple CRPs in comparison to a single CRP for mitigating the risk of RD remains uncertain.

Moreover, previous studies predominantly employed manual repositioning, which could not ensure consistency in the repositioning procedures. Mechanical rotational chair-assisted multiple CRPs, as employed in the present study, facilitate standardized CRP treatment for BPPV, circumventing discrepancies in physical maneuvers executed by various physicians with respect to angle and velocity (8–11).

To the best of our knowledge, no existing research has appraised the advantages, drawbacks, and potential hazards of residual dizziness (RD) associated with mechanical rotational chair-assisted multiple CRPs. Our objective was to evaluate the effectiveness of multiple CRPs in treating BPPV by employing a BPPV diagnosis and treatment system.

## Materials and methods

### Ethics approval

This study received approval from the Rizhao People's Hospital Ethics Committee (Ethics Approval Number: 2023-CG-01). Owing to the retrospective nature of the investigation, informed consent was not necessitated.

### Participants

**Figure 1:**
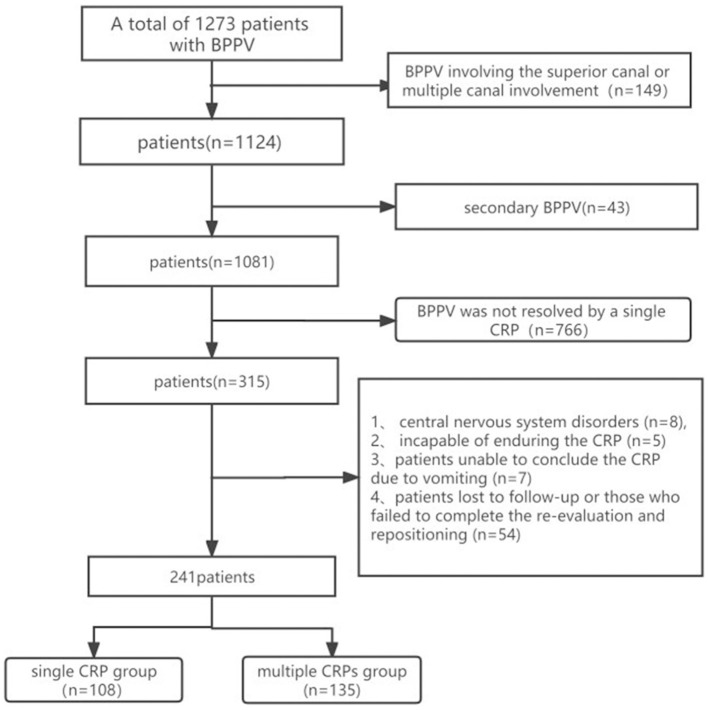
Inclusion and exclusion flow diagram.

We conducted a retrospective analysis of data from 1,273 BPPV patients treated at the People's Hospital of Rizhao between March 2017 and November 2021. Ultimately, 241 patients were incorporated into the study. The inclusion criteria were as follows: (1) those diagnosed with BPPV according to the Bárány Institute's latest BPPV diagnostic criteria (12), (2) BPPV involving the posterior canal (PC) or horizontal canal (HC), and (3) BPPV not resolved by a single CRP. The exclusion criteria were as follows: (1) BPPV involving the superior canal or multiple canal involvement, (2) secondary BPPV (BPPV arising as a secondary condition resulting from another primary medical condition or event, such as a head trauma, ear surgery, or certain diseases of the inner ear), (3) central nervous system impairment symptoms, (4) patients unable to conclude the CRP on account of vomiting or those demonstrating intolerance to the CRP, and (5) patients who were lost to follow-up or those who failed to complete the necessary re-evaluation and repositioning process. The consort flow diagram is depicted in the subsequent schematic representation (Figure 1).

### Protocol

In the single CRP group, we included 106 participants from March 2017 to June 2019. Each participant underwent a single CRP in one session, and a subsequent positive Dix–Hallpike (DH) or roll test performed 20 min later revealed no alleviation of BPPV symptoms. To improve the resolution rate of vertigo, we began exploring multiple CRPs starting from June 2019. The multiple CRP group included 135 participants selected from June 2019 to November 2021. Subsequent to the initial CRP yielding a positive D-H or roll test result, additional CRPs were administered, maintaining an interval of 20 min between each session, with a maximum of three treatment sessions.

In cases where vertigo and nystagmus remained unresolved, patients underwent the D-H or roll test on day 1, day 4, and day 7 after the initial treatment to evaluate the effectiveness of treatment. Subsequently, CRPs were diligently administered. The success of treatment was determined based on the results of the DH or roll tests administered subsequent to the final CRP at each follow-up visit. Concurrently, the cumulative relief rate was calculated. However, in cases where vertigo and nystagmus ceased, consecutive evaluation was not performed, necessitating only outpatient or telephone follow-ups.

Certain patients, despite the cessation of nystagmus and vertigo after undergoing D-H or roll test, reported persistent vertigo during the follow-ups on the 4th or 7th days. In such instances, a further D-H or roll test was carried out, and if found positive, it was defined as unresolved BPPV. It is important to underscore the difficulty in discerning whether these patients are unimproved or are experiencing a re-emergence of BPPV. However, the probability of a fresh occurrence of BPPV is less likely than that of unresolved BPPV. Consequently, these cases are uniformly considered unresolved BPPV. In some patients, the DH test showed positive results after repositioning; however, it turned negative in the DH test during subsequent follow-ups (1 day, 4 days, or 1 week). This indicates spontaneous remission of BPPV in these cases. The treatment success time for these patients is still defined as the day of the follow-up.

For patients experiencing vomiting, we typically recommend a rest period of 30 min, followed by the administration of promethazin for the antiemetic effect before proceeding with further repositioning treatment. The majority of patients do not experience further vomiting during subsequent repositioning. Patients who could not tolerate vomiting were ultimately not included in the study, as depicted in the consort flow diagram.

Residual dizziness (RD), despite the lack of a universally recognized definition, is characterized by persistent or intermittent sensations of head heaviness, imbalance, or non-specific dizziness (13). Therapeutic success was determined by the resolution of both vertigo and nystagmus in patients. Evaluations for RD commenced from the day after the successful intervention and thereafter on the 3rd day, the 1st week, and subsequently on a weekly basis. These assessments were facilitated through direct outpatient consultations or telephonic follow-ups, utilizing verbal communication. Follow-ups were continued until the cessation of RD. We utilized RD incidence and duration to evaluate the impact of RD.

### BPPV diagnosis and treatment system

We used an automated 3D rotational chair from Byrons Medical Science & Technique Inc., along with a video eye mask and workstation. The chair can rotate 360° in all directions, and the mask helps visualize nystagmus. The patient's head was securely fastened with a strap, prohibiting free movement, and consequently moved along with the chair. This system can consistently mimic CRPs. The Dix–Hallpike test, used to diagnose PC-BPPV, involves torsional nystagmus where the eyes move toward the lower ear and vertically upward, with symptoms typically lasting less than a minute. The supine roll test is for diagnosing HC-BPPV. The patient is rolled with their head turned sideways, inducing horizontal nystagmus toward the lowermost ear. This typically lasts less than a minute. In the case of cupulolithiasis of the horizontal canal (HC-BPPV-cu), the same test induces nystagmus toward the uppermost ear, usually lasting longer than a minute. The system was primarily manipulated by the operators, who adjudicated the involved semicircular canals and laterality based on the nystagmus direction, speed, and intensity information displayed on the video goggles.

### Treatment method

Participants with PC-BPPV underwent Epley maneuvers. To illustrate, let us take left PC-BPPV as an example: (1) While seated, the participant is rotated to the left by 45° and maintains this position for 1 s. (2) The body is then tilted backward at an angle of 120° with a velocity of 120°/s for a duration of 60 s. (3) The head and body are turned to the right by 90° at a velocity of 90°/s, maintaining this position for 60 s. (4) The head and body continue to turn to the right by another 90° at the same acceleration, again holding this position for 60 s. (5) Finally, the participant returns to the initial seated position. For lesions on the right side, the above steps should be performed in reverse, while keeping the parameters unchanged.

Patients with HC-BPPV received the barbecue maneuver. After being positioned in a supine position, the device rotates the patient four times to the affected side at an angle and velocity of 90°/s, completing a full 360° rotation. Each position is maintained for at least 60 s. Finally, the patient is slowly and carefully returned to the seated position.

Patients suffering from HC-BPPV (cu) are treated using the Gufoni maneuver. A mechanical rotational chair rapidly moves the individual to a lateral position, with the affected ear facing downward, and maintains this position for 60 s. Subsequently, the chair rotates the individual's head upward by 45 degrees at a velocity of 90°/s, directing the nose toward the ceiling. This position is maintained for the same duration of 60 s. Finally, the chair slowly returns the individual to an upright seated position. After preserving this position for another 60 s, the patient is gently assisted back to an upright sitting position. A roll test is performed 10 min later, with a negative result indicating successful repositioning. However, if the direction of the nystagmus changes to geotropic, the barbecue maneuver is employed for the repositioning of the HC.

### Statistical analysis

We executed the statistical analysis employing SPSS version 18.0 (IBM Corp., Armonk, NY, USA). The results were deemed statistically significant when the *P*-value is <0.05. The independent sample *t*-test was employed to compare age differences between the two groups. The chi-square test was utilized for categorical variables to evaluate the disparities between the groups in terms of gender, affected canal, affected side, resolution of positional nystagmus and vertigo, adverse effects, and residual dizziness. Lastly, the Mann–Whitney U-test, a non-parametric test, was applied to compare the groups concerning variables such as vertigo duration (in days), duration of vertigo before treatment (in days), and number of CRPs. The duration of RD was appraised using Kaplan–Meier survival analysis, with survival time defined as the number of days from the onset to the conclusion of RD.

## Results

The baseline variables (age, gender, affected canal, and affected side) exhibited no significant differences between the two groups (*p* > 0.05). The duration of vertigo was defined as the period from the onset of vertigo to its cessation. The duration of vertigo persisted for a more extended period in the single CRP group compared to the multiple CRP group (*P* = 0.001). The duration of vertigo before treatment, defined as the span of time from the onset of vertigo until the commencement of the CRP, showed no difference between the two groups (*P* = 0.058). Additionally, the number of CRPs was lesser in the single CRP group than in the multiple CRP group (*P* = 0.007) (Table 1).

**Table 1:** Baseline features of the study subjects.

	**Total (241)**	**Single CRP group (106)**	**Multiple CRP group (135)**	***P***
Age (mean ± SD) (25–81 years)	60.7 ± 11.8	60.5 ± 10.9	61.0 ± 12.8	0.747^a^
Gender				0.571^b^
Male	82	34	48	
Female	159	72	87	
Affected canal				0.621^b^
Posterior	155	70	85	
Horizontal	86	36	50	
Affected side (right/left)				0.402^b^
Right	117	50	67	
Left	124	56	68	
Duration of vertigo, days; Mdn (IQR)	5 (3-9)	5 (3.75-10)	5 (2-7)	0.001^c^
Duration of vertigo before treatment, days; Mdn (IQR)	4 (2-6)	4 (2-6.25)	4 (2-6)	0.058^c^
Number of CRPs; Mdn (IQR)	2 (2-4)	2 (2-3)	2 (2-4)	0.007^c^

^a^Independent samples *t*-test,

^b^chi-square test,

^c^Mann–Whitney U-test.

IQR, interquartile range; SD, standard deviation; the duration of vertigo, the period from the onset of vertigo to its cessation; duration of vertigo before treatment, the span of time from the onset of vertigo until the commencement of the CRP.

Pertaining to the resolution of positional nystagmus and vertigo (Table 2), the multiple CRP group exhibited a resolution rate of 50.4% immediately following the second CRP and 63.7% immediately after the third CRP. On day 1, the resolution rate was notably higher in the multiple CRP group (85.2%) compared to the single CRP group (55.7%) (*P* < 0.001). On day 4, the cumulative resolution rate was significantly elevated in the multiple CRP group (91.9%) relative to the single CRP group (75.5%) (*P* = 0.001). On day 7, the cumulative resolution rate reached 94.8% in the multiple CRP group and 93.4% in the single CRP group (*P* = 0.871), with no significant difference observed. A total of 13 patients in the single CRP group experienced spontaneous remission compared to 7 patients in the multiple CRP group (12.3% vs. 5.1%, *P* = 0.048).

**Table 2:** Resolution of positional nystagmus and vertigo (cumulative percentage).

	**Single CRP group**	**Multiple CRP group**	**χ^2^ test *P***
Resolution immediately after second CRP, *n* (%)		68/135 (50.4%)	
Resolution immediately after third CRP, *n* (%)		86/135 (63.7%)	
Resolution on day 1 after treatment, *n* (%)	59/106 (55.7%)	115/135 (85.2%)	*P* < 0.001
Resolution on day 4 after treatment, *n* (%)	80/106 (75.5%)	124/135 (91.9%)	0.001
Resolution on day 7 after treatment, *n* (%)	100/106 (93.4%)	128/135 (94.8%)	0.871

The success of treatment was determined based on the results of the DH or Roll tests administered subsequent to the final CRP at each follow-up visit. Concurrently, the cumulative relief rate was calculated.

In relation to the adverse effects of CRP (Table 3), 5.2% of participants in the multiple CRP group developed CS compared to 3.8% in the single CRP group; however, the difference was not statistically significant (*P* = 0.602). Within the multiple CRP group, CS occurred in 3% (4/135) of PC-BPPV patients, involving conversions between the posterior canal (PC) and the horizontal canal (HC) (2.2%, 3/135) as well as between the PC and the anterior canal (AC) (0.7%, 1/135). CS involving the HC encompassed transitions from the HC to the PC (1.48%, 2/135) and from the HC to AC (0.74%, 1/135). In the single CRP group, CS occurred in 3.7% (4/106) of PC-BPPV patients, involving conversions between PC and HC (2.2%, 2/106), including from HC to PC (1.5%, 2/135). The sample size for CS types was insufficient for conducting statistical analysis. No significant differences were observed between the single and multiple CRP groups regarding falls (10.3% vs. 8.9%; *P* = 0.696). Nevertheless, a significant difference between the single and multiple CRP groups was detected in the incidence of vomiting (6.6% vs. 14.8%; *P* = 0.015).

**Table 3:** Adverse effects.

	**Total**	**Single CRP group**	**Multiple CRP group**	**χ^2^ test *P***
Canal conversion	11/241 (4.6%)	4/106 (3.8%)	7/135 (5.2%)	0.602
Vomiting	30/241 (12.4%)	7/106 (6.6%)	23/135 (14.8%)	0.015
Fall	23/241 (9.5%)	11/106 (10.3%)	12/135 (8.9%)	0.696

Following CRP, distinct between-group disparities were observed, all favoring the multiple CRP group, in terms of head heaviness, imbalance, and non-specific dizziness rates (*P* < 0.05). For participants (all requiring >1 maneuver, according to the study eligibility criteria), the RD rate was 41.5% in the multiple CRP group as opposed to 57.5% in the single CRP group (*P* = 0.013) (Table 4).

**Table 4:** Residual dizziness.

	**Total**	**Single CRP group**	**Multiple CRP group**	**χ^2^ test *P***
Patient-reported RD	117/241 (48.5%)	61/106 (57.5%)	56/135 (41.5%)	0.013
Head heaviness	65/241 (27.0%)	37/106 (34.9%)	28/135 (20.7%)	0.014
Imbalance	81/241 (33.6%)	45/106 (42.5%)	36/135 (26.7%)	0.010
Non-specific dizziness	94/241 (39.0%)	50/106 (47.2%)	44/135 (32.6%)	0.021

**Figure 2:**
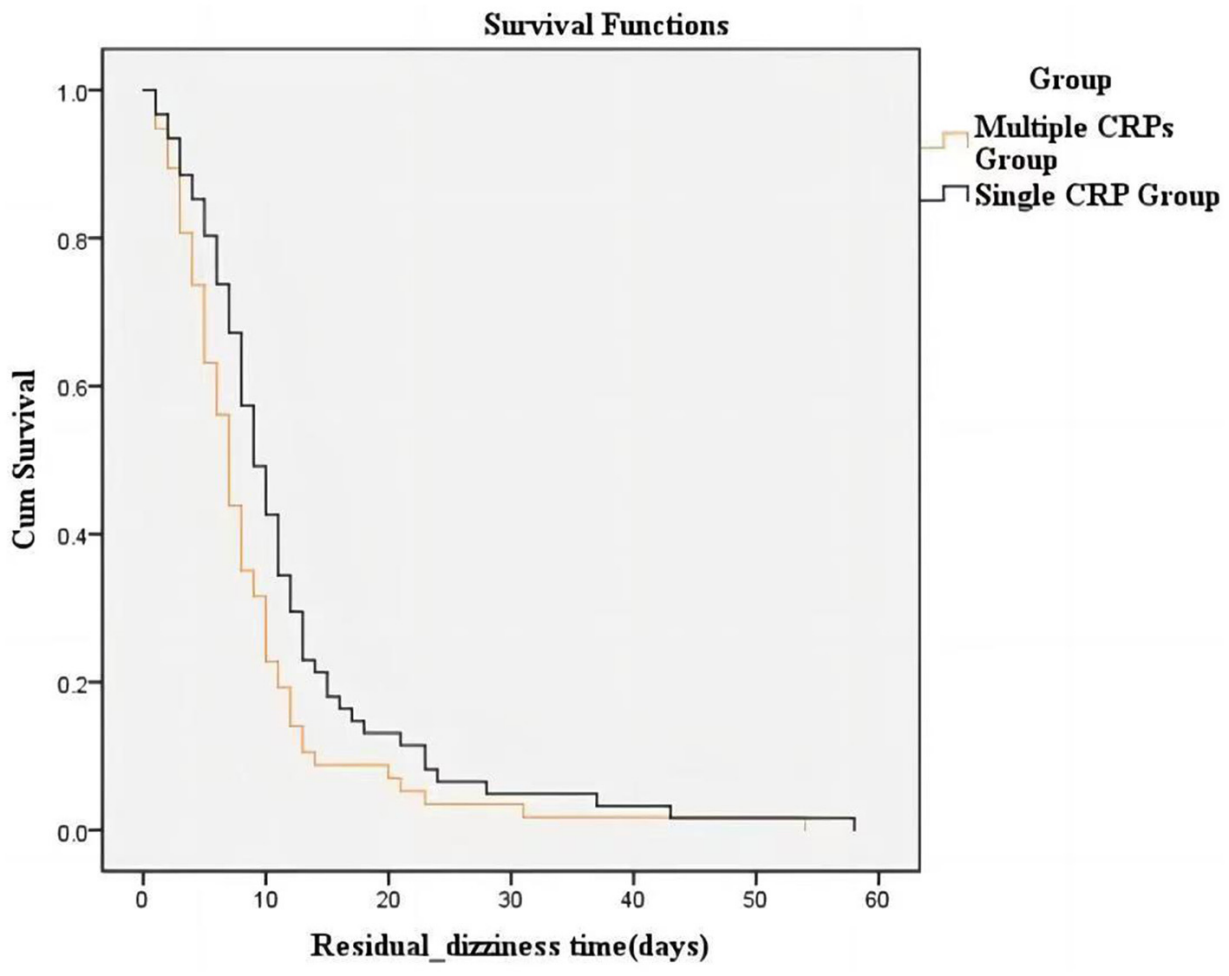
Cumulative proportion of participants with RD over the 3-month follow-up in the two groups. Kaplan–Meier survival curve was used to compare the duration of RD between the single CRP group and multiple CRP groups in patients with BPPV. The survival curve demonstrates the probability of experiencing residual vertigo over time for each group. The curve for the multiple repositioning group consistently remains above the curve for the single CRP group, indicating a higher probability of faster resolution of vertigo symptoms in the multiple CRP group. The log-rank test was performed to assess the statistical significance of the difference between the two groups (*P* = 0.025).

RD abated within 20 days for the majority of participants, and none experienced dizziness after 3 months. The duration of RD was shorter in the multiple CRP group compared to the single CRP group (*P* = 0.025) (Figure 2).

## Discussion

In this retrospective study, we found that for patients with BPPV, multiple CRPs performed during a single consultation with the assistance of the mechanical rotational chair are more advantageous than a single CRP. Specifically, multiple CRPs potentially provide faster relief for positional vertigo without additional side effects and reduce the incidence and duration of RD.

Although guidelines recognize traditional manual CRP as an efficacious approach to treating BPPV (1), it lacks standardization and consistency. Patients' apprehension of dizziness might undermine the uniformity of posture and repositioning angles, while diverse repositioning practices among physicians lead to disparities in angles, rotation speeds, and interval durations, potentially affecting study outcomes. In our research, we employed a mechanical rotational chair-assisted CRP, ensuring procedural consistency regarding angles, rotation speeds, and interval times. The effectiveness of mechanical rotational chair-assisted repositioning has been substantiated in previous studies. Nakayama et al. (14) utilized a power-driven multiaxial repositioning chair to address several BPPV variants using a single CRP in one session, achieving favorable treatment results. Lou et al. (8) employed a multiaxial positioning device to treat PC-BPPV, following a maximum of three sessions (one CRP per session) over 2 weeks, and 97% of patients experienced relief from vertigo and nystagmus.

Numerous studies (4–6, 15) have indicated that the efficacy of multiple CRPs across various sessions (measured by the resolution rate of positional vertigo and nystagmus) is equivalent to or surpasses that of a single CRP. A systematic review disclosed a considerable variation in CRP resolution rates, particularly for multiple CRPs, ranging from 68% to 90% following one session, 40% to 100% after two sessions, and 67% to 98% after three sessions (16). Nonetheless, a randomized controlled trial ascertained that single-cycle CRP was as effective as multiple-cycle CRP, exhibiting a decreased incidence of complications and a diminished treatment duration (4). Previous investigations either utilized manual repositioning, lacked a control group, or exclusively concentrated on the efficacy of multiple repositioning maneuvers, neglecting a systematic evaluation of potential side effects associated with escalating repositioning frequency. For example, multiple repositioning might heighten the probability of canal conversion, intensifying and prolonging dizziness (7). Augmenting repositioning frequency could give rise to adverse effects such as nausea and vomiting, consequently disrupting treatment. Conversely, residual dizziness, a post-effect of BPPV, has not been methodically appraised in earlier studies. In our investigation, we undertook a thorough and systematic assessment of the risk-benefit ratio of machine-assisted multiple BPPV repositioning maneuvers and RD after repositioning, offering invaluable guidance for BPPV treatment.

Regarding the success rate of multiple CRPs, a discrepancy in the resolution rate on days 1 and 4 emerged between the single and multiple CRP groups (63.2% vs. 85.1%, 75.5% vs. 91.9%; *P* < 0.05) though not on day 7. Kaewsiri Isaradisaikul et al. established that the negative rate of the DH test within the initial week for the single repositioning and multiple repositioning cohorts was 76.9% and 76.7%, respectively, which is inferior to our study. This might be attributable to our repeated implementation of CRP treatment on the 1st day, 4th day, and within 1 week after the initial treatment. The multiple CRPs performed during the initial session may also have significantly contributed to the outcomes. In this investigation, the curtailed CRP treatment duration at the 1st and 4th days after the initial treatment could be a contributing factor to the diminished DH test negative rate (4). In conclusion, executing multiple CRPs within a single session is more expedient than a single CRP as it reduces the duration of vertigo and alleviates undue distress stemming from BPPV symptoms.

Regarding side effects associated with multiple CRPs, we compared the adverse effects between the two groups to ascertain whether multiple CRPs lead to a higher rate of adverse effects, such as vomiting, falls, and particularly CS. The rate of vomiting was lower in the single CRP group than in the multiple CRP group. However, nearly all participants recovered promptly. Symptoms of nausea tend to self-resolve, while vomiting can be rapidly alleviated with promethazin administration. No other severe complications resulted from multiple CRPs. The presence of dizziness in the elderly is a strong predictor of falls, which is the leading cause of accidental death in people older than 65 years (17). Previous research verified that CRP in the elderly significantly reduced the number of falls (18). In our study, although multiple repositioning treatments offered a faster relief rate for benign paroxysmal positional vertigo, we identified no significant difference in the incidence of falls between the two groups. Our study population, which was not restricted to the older population, may have influenced the results.

CS constitutes a primary concern in relation to adverse effects. Otoliths may migrate to an alternate canal or re-enter the originating canal because of CRP. Foster et al. reported that ~16% of BPPV patients encountered CS when the Dix–Hallpike test was performed 15 min after the initial CRP (7). Wu et al. showed the CS rate was 4.6% (19). Both studies employed manual CRPs. In our study, the rate of CS was 5.1% in the multiple CRP group compared to 3.8% in the single CRP group (*P* > 0.05). Although the CS rate was higher in the multiple CRP group than in the single CRP group, the difference was not statistically significant. Lee et al. investigated the incidence of CS in patients with PC-BPPV and horizontal canal-BPPV HC-BPPV, finding a significant association between CS and the use of multiple CRP sessions (20). However, our research focused on BPPV patients who underwent multiple repositioning treatments. Incorporating BPPV patients who achieved successful treatment through a solitary repositioning procedure into a more expansive sample could potentially yield a lower CS rate. Dan et al. also disclosed a reduced canal conversion rate (2/132, 1.5%) among patients undergoing computer-assisted CRP treatment (9). The diminished canal conversion rate observed in our study may also be ascribed to the use of a computer-controlled automated CRP, which affords standardized CRP, concurrent monitoring of nystagmus, and head position, thus diminishing the likelihood of CS.

RD constitutes a common symptom encountered in clinical practice. Especially among elderly individuals and patients with psychiatric disorders, RD may lead to an increased risk of falls, restricted activity, and associated social and economic burdens (18). In previous studies, the incidence of RD following CRP has been reported to range between 36.6% and 61% (21). Vaduva et al. demonstrated that among patients with BPPV, those necessitating multiple CRPs (≥2 maneuvers−132 patients) exhibited a significantly higher incidence of RD relative to those necessitating merely a single CRP (1 maneuver−229 patients) (50% vs. 17.9%; *p* < 0.0001) (22). As per a meta-analysis study, the existence of a correlation between the number of CRPs performed and the incidence of RD remains inconclusive (23). In our study, all included patients underwent more than one repositioning, resulting in an overall residual dizziness incidence rate of 48.5%, in alignment with other studies (22, 23). In our study, the group subjected to multiple repositioning demonstrated a higher number of repositioning interventions as well as a lower incidence and duration of residual dizziness. Nonetheless, this does not necessarily imply a negative correlation between the frequency of repositioning and the incidence of residual dizziness. The disparity in dizziness recovery times between the two groups may engender varying rates of spontaneous resolution.

Nonetheless, in our study, relative to the single CRP group, the multiple CRP group displayed a significantly diminished incidence rate and duration. This finding implies that multiple CRP treatments may positively influence the reduction of both the occurrence and duration of RD. Di Girolamo et al. postulated that incomplete repositioning may result in residual otoconial debris sufficient to provoke mild vertigo (24, 25). Multiple CRPs may lead to less of this residual otoconial debris, leading to a lower incidence and duration of RD than after a single CRP.

A previous study reported that the duration of vertigo before CRP was related to the presence of RD (13, 23). Faralli posited that the initial asymmetry of peripheral vestibular function caused by BPPV can induce novel central adaptations. As the otoconial particles remain suspended in the endolymph for extended periods, their adaptive capacity is heightened, rendering the brain incapable of rapid adjustment following the successful execution of canalith repositioning procedures (CRPs), consequently leading to RD (26). Compared to the multiple CRP group, the single CRP group increases the duration of positional vertigo, and a slowed BPPV recovery necessitates more time for central adaptation after particle repositioning. This is consistent with our research findings, where the duration of vertigo in the multiple CRP group was lower than that in the single CRPs group. Teggi et al. emphasized that a prolonged duration of vertigo intensifies the degree of anxiety. Subsequently, such anxiety can escalate the incidence of residual dizziness (27). Hence, multiple CRPs may result in a reduced RD rate (41.5% vs. 57.5% in the multiple and single CRP groups, respectively, in our study).

Our study, however, is not without its limitations. First, our research is retrospective in design, and potential selection bias cannot be ruled out. Second, as the study was conducted in a single hospital, the relatively small sample size may not comprehensively reflect the conditions of all BPPV patients. Moreover, our research did not involve long-term follow-up of patients, thereby precluding any definitive conclusions about the long-term effects and complications of CRP treatment.

## Conclusion

For patients with BPPV, multiple CRPs performed during a single consultation with the assistance of the mechanical rotational chair are more advantageous than a single CRP. This is because they potentially provide faster relief for positional vertigo without additional side effects and reduce the incidence and duration of RD.

## Data availability statement

The raw data supporting the conclusions of this article will be made available by the authors, without undue reservation.

## Ethics statement

The studies involving human participants were reviewed and approved by the Rizhao People's Hospital Ethics Committee (Ethics Approval Number: 2023-CG-01). Written informed consent for participation was not required for this study in accordance with the national legislation and the institutional requirements.

## Author contributions

HZ designed the study, collected the data, analyzed the data, and wrote the manuscript. MZ contributed to the study design, assisted with data collection and analysis, and provided critical revisions to the manuscript. All authors have read and approved the final version of the manuscript.
